# Influence of *Enterocytozoon bieneusi* Infection on Child Growth: A Secondary Analysis of the MAL-ED Birth Cohort Study

**DOI:** 10.4269/ajtmh.23-0895

**Published:** 2025-01-28

**Authors:** Md Ahshanul Haque, Shaumik Islam, Syed Jayedul Bashar, Abu Sayem Mirza Md. Hasibur Rahman, A. S. G. Faruque, Tahmeed Ahmed, Mustafa Mahfuz

**Affiliations:** ^1^ Nutrition Research Division, icddr,b, Dhaka, Bangladesh;; ^2^ Infectious Diseases Division, icddr,b, Dhaka, Bangladesh

## Abstract

Malnutrition in the early days of life is a global public health concern that affects children’s growth. It results from a variety of factors, including pathogenic infections. *Enterocytozoon bieneusi* is a microsporidian parasite that can cause diarrhea and malnutrition in children. The study aimed to assess the impact of *E. bieneusi* on child growth. The MAL-ED study, a multicountry birth cohort research project, investigated the relationship between enteric infections and malnutrition in participating children from eight countries. A customized real-time polymerase chain reaction-based TaqMan array card was used in this study to identify enteropathogens from stool samples, where *E. bieneusi* was one of the target pathogens. The impacts of *E. bieneusi* infection on growth measures were assessed. Mixed-effect linear models were used to investigate the relationship between *E. bieneusi* and growth outcomes, including length-for-age (LAZ), weight-for-age (WAZ), and weight-for-length (WLZ) Z scores. At the endpoint (last month of measurement), the infected group had significantly lower scores than the noninfected group for all outcomes. The adjusted difference-in-difference (D-in-D) values were −0.53 (95% CI: −0.67 to −0.38) for LAZ, −0.38 (95% CI: −0.52 to −0.23) for WAZ, and −0.22 (95% CI: −0.38 to −0.06) for WLZ. *Enterocytozoon bieneusi* infection has been identified as a factor associated with reduced linear growth, weight gain, and weight gain relative to linear growth in children, underscoring the importance of treating this infection.

## INTRODUCTION

Childhood malnutrition is a significant public health challenge that affects children globally. It can result in stunting, wasting, and underweight, each of which measures chronic or acute nutritional deficiency. A recent Bangladesh Demographic and Health Survey suggested that among children under 5 years of age, 24% are stunted, 22% are underweight, and 11% are wasted.^1^ Maternal health, food insecurity, household size, dietary diversity, sex of the household head, and presence of morbidity are some of the factors that impact childhood nutrition in developing nations.^2^^–^^6^

Pathogenic infections are the most important factors that are influenced by malnutrition.^7^^,^^8^ *Enterocytozoon bieneusi*, an obligate intracellular parasite from the Microsporidia division, infects intestinal epithelial cells in humans, pigs, and other mammals. In the late 1920s, *E. bieneusi*, the most widely known microsporidian parasite, was identified as a spore-forming eukaryote that can cause diarrhea in humans, particularly in immunocompromised individuals, but *E. bieneusi* infections were not associated with lower survival despite the presence of low CD4 counts in people without antiretroviral therapies.^9^^,^^10^ Serious malabsorption of vitamins, micronutrients, carbohydrates, and fats, along with decreased serum folate and zinc levels, is found in patients with intestinal microsporidiosis unlike in *E. bieneusi*-negative patients with persistent diarrhea.^11^ A small intestinal lesion with minor villus atrophy and crypt hyperplasia was observed in half of the individuals with microsporidia but not in pathogen-negative controls with diarrhea.^12^

Immunocompetent people frequently experience persistent asymptomatic microsporidian infections, whereas immunocompromised hosts predominantly experience fatal infections.^13^ *Enterocytozoon bieneusi* has a life cycle involving a proliferative stage and a sporogony stage that produce infective spores. Protected by a spore coat, these spores survive outside the host and can be transmitted through water or food. The spore coat aids in adherence and interaction with host cells.^14^^–^^16^ The polar tube, a key infection apparatus, allows the spores to infect host cells by delivering the sporoplasm into the host’s cytoplasm. The exact mechanism of spore germination varies among species and involves activation, increased osmotic pressure, and ejection of the polar tube.^17^^–^^21^

Albendazole, a benzimidazole that binds to β-tubulin, is used to treat microsporidiosis and has an action against many species of microsporidia, but it is ineffective against *E. bieneusi* infections. *Enterocytozoon bieneusi* has tubulin genes that encode amino acid sequences linked to albendazole resistance.^22^^,^^23^ AIDS patients were the first reported to experience diarrhea brought on by *E. bieneusi* microsporidiosis, in 1985.^24^ In patients who have undergone organ transplantation, nitazoxanide therapy has been successful in stopping diarrhea brought on by *E. bieneusi*; however, this effect may rely on the patient’s immune status, as this medication was only marginally effective in AIDS patients with low CD4 counts.^25^

In children, diarrhea caused by *E. bieneusi* can result in dehydration and malabsorption of nutrients, leading to malnutrition and impaired growth.^26^ It causes diarrhea and can easily spread, affecting a variety of hosts, including pigs, humans, and other mammals. The pathogen can be studied through techniques such as transmission electron microscopy, light microscopy, polymerase chain reaction (PCR), and immunofluorescence and can be cultured for a short period of time.

*Enterocytozoon bieneusi* is highly contagious and spreads through the excretion of infective fragments.^27^ Although the impact of *E. bieneusi* on childhood malnutrition is yet to be fully understood, it has been suggested that the pathogen may play a role in a lower rate of weight gain that results in stunting, wasting, and underweight.^28^ The purpose of this paper is to assess the country-specific association between *E. bieneusi* infection and all forms of childhood malnutrition. This study aims to fill the gap in the literature and provide a solid background for a proper understanding of the impact of *E. bieneusi* on the nutritional status of children. The results of this study will help researchers develop a plan for efficient public health interventions aimed at reducing childhood malnutrition and improving child health globally.

## MATERIALS AND METHODS

### Data source.

Data were derived for this paper from the MAL-ED (Etiology, Risk Factors, and Interactions of Enteric Infections and Malnutrition and the Consequences for Child Health Development) multicountry birth cohort study. The MAL-ED study was a multicountry birth cohort research that aimed to investigate the relationship between enteric infections and malnutrition in children.^29^ We conducted the study in eight different geographic locations, including Bangladesh, India, Nepal, Pakistan, South Africa, Tanzania, Brazil, and Peru. We excluded children from the Pakistan site for growth analysis because of the bias noted in a subset of length measurements at this site. The study enrolled over 200 newborns per site from November 2009 to February 2012 from the community within 17 days of birth in each site and involved twice-weekly home visits to the participants. The inclusion criteria for the study included maternal age of 16 years or older, singleton pregnancy, weight at birth or enrollment greater than 1.5 kg, and intention of the family to stay in the study area for at least 6 months. The study obtained written informed consent from the parents or caregivers and followed ethical requirements set by the local regulatory authorities. We assembled the study participants from various locations and had their anthropometric data, birth history, household demographics, and maternal factors recorded at the time of recruitment.^30^

### Variables under study.

The children’s birth, household, maternal, and anthropometric data were collected. Anthropometric measurements were obtained using length measurements instead of height, considering the age range, below 24 months, of the children and practical aspects of the measurement process. Length measurements were taken with the child lying supine on an anthropometry tool (Seca GmbH & Co. KG, Hamburg, Germany) and conducted by trained personnel to ensure accuracy and consistency. Measurements were performed monthly on all children using standardized procedures and collected every month with standard scales and the 2006 WHO guidelines. The children’s growth was assessed through the calculation of length-for-age Z scores (LAZ), weight-for-age Z scores (WAZ), and weight-for-length Z scores (WLZ) as outcome variables. The Z-score scale, calculated as (observed value − average value of the reference population)/standard deviation value of the reference population, is linear, and therefore a fixed interval of Z-scores has a fixed length difference in centimeters for all children of the same age.^31^ The exposure variable in this analysis was *E. bieneusi* infection, which was extracted from laboratory results. The laboratory techniques used to test the stool samples collected monthly by the community research staff were identical and synchronized among all participating laboratories at each study site. The samples underwent total nucleic acid extraction and standard protocols to detect 29 enteropathogens in a single sample.^32^ This was done using a customized real-time PCR-based TaqMan array card. The analytical limit was set at 35 threshold cycles, and a value of less than 35 was considered positive for *E. bieneusi* infection.^30^ The other covariates were the child’s sex, household socioeconomic status (SES), and maternal height, mother with fewer than three living children and environmental enteric dysfunction (EED) scores,^33^^,^^34^ and site. The water/sanitation, assets, maternal education, and income (WAMI) index was treated as SES. The composite measure called the WAMI index, based on maternal education, improved water and sanitation, eight selected assets, and household income, was established for each site.^35^ The other pathogens, *Aeromonas*, *Bacteroides fragilis, Campylobacter jejuni/coli, Clostridium difficile*, Enteroaggregative *Escherichia coli*, Typical Enteropathogenic *Escherichia coli*, Shiga Toxin-producing *Escherichia coli*, Enterotoxigenic *Escherichia coli*, *Blastocystis* sp., *Cryptosporidium*, *Giardia*, adenovirus 40/41, astrovirus, rotavirus, sapovirus, and norovirus were included as copathogens in the statistical analysis related to a child’s growth.

**Figure 1.**
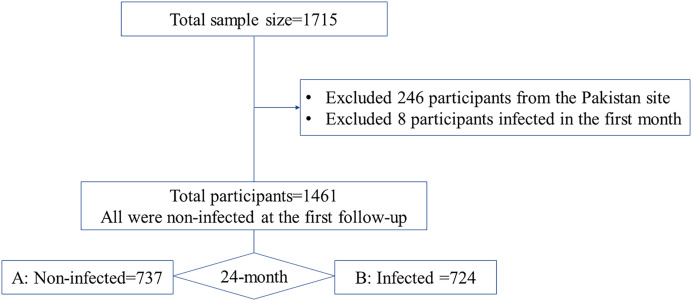
Time points at which measurements were taken: the first-month measurement (baseline) and the last-month measurement (endpoint).

From the 24-month follow-up birth cohort study, we defined baseline and endpoint measurements and the *E. bieneusi* infection status of children as the exposure variable. The study includes two groups: group A, which comprised children that remained noninfected throughout the 24-month follow-up period, and group B, which comprised children that were noninfected at the first follow-up but became infected with *E. bieneusi* at least once during the 24 months of follow-up. Figure 1 shows the time points at which measurements were taken: the first-month measurement (baseline) and the last-month measurement (endpoint). The figure provides a clear description of the infection status of each group at each time point, with “infected” indicating that the child had an *E. bieneusi* infection in at least 1 month during the follow-up period and “noninfected” indicating that the child did not have an *E. bieneusi* infection during the follow-up period.

## STATISTICAL ANALYSES

The analysis was done using Stata software. Quantitative variables were summarized using the mean and SD, whereas qualitative variables were summarized with frequency and proportion. The study used line graphs to visualize *E. bieneusi* infection status over time. The Kaplan-Meier survival graph was used to visualize the incidence of *E. bieneusi* infection over time due to several factors. This allowed for the comparison of probability of infection between different groups and the identification of any significant differences. A mixed-effect linear model was used at the site level to assess the relationship between *E. bieneusi* and a child’s LAZ, WAZ, and WLZ after adjustment for factors such as sex, WAMI index, maternal height, number of surviving children, and copathogens, which were selected based on a literature review as well as the bivariate analyses. The analyses were conducted simultaneously at baseline and endpoint measurements. The impact of *E. bieneusi* on outcomes was measured using difference-in-difference analysis (D-in-D). To estimate the D-in-D parameter, we used the interaction term between the group variable (group A = 0; group B = 1) and time point (baseline = 0; endpoint = 1) in the mixed-effect model. Here, group A means “at both baseline and endpoint, there was no infection with *E. bieneusi*” and group B means “*E. bieneusi* infection was not present at baseline but developed during the 24-month follow-up period.”

## RESULTS

### General characteristics.

#### Status of E. bieneusi infection.

**Figure 2.**
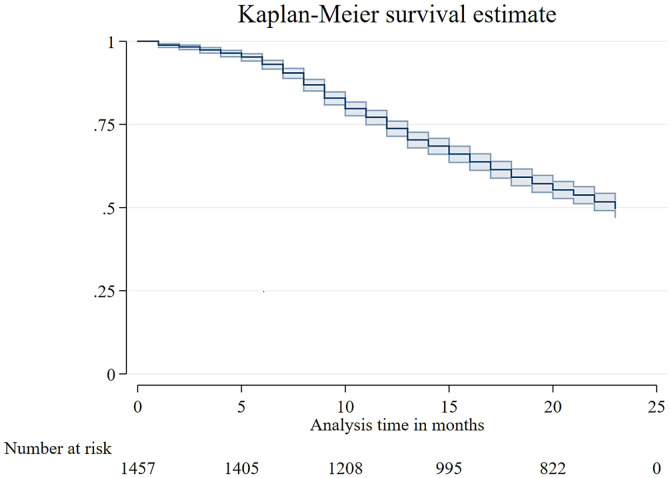
Kaplan-Meier survival plot of *Enterocytozoon bieneusi* infection.

A total of 1,715 participants who completed follow-up for 24 months contributed 34,622 surveillance stool samples tested for *E*. *bieneusi* by quantitative PCR. Data from 246 participants at the Pakistan site were excluded. Additionally, 8 participants infected in the first month were excluded. Finally, this paper analyzed a total of 1,461 participants, of whom 724 (49.5%) (Figure 2) children were found to be infected with *E. bieneusi* and the remaining 737 were noninfected all through the 24-month follow-up.

The general characteristics of the study participants are presented in Table 1. The sex of the participants was almost equally distributed across all the sites. Bangladesh had the highest average number of days of exclusive breastfeeding (143.2 ± 42.7), whereas South Africa had the lowest. Length-for-age Z scores at the first follow-up were highest in Brazil (−0.3) and lowest in India and Peru (−1.2). Weight-for-age Z scores at the first follow-up were highest in Brazil (0.4) and lowest in India (−1.4). Weight-for-length Z scores at the first follow-up were highest in Brazil (0.9) and lowest in India (−0.8). At 24 months, LAZ scores were highest in Brazil (0) and lowest in Tanzania (−2.7), with WAZ scores highest in Brazil (0.4) and lowest in India (−1.7). Weight-for-length Z scores at 24 months were highest in Brazil and South Africa (0.5) and lowest in India (−0.9). Maternal age was highest in Tanzania (29.1 year) and lowest in India (23.9 years), whereas maternal height was greatest in South Africa (158.7 cm) and lowest in Bangladesh (149.0 cm). The percentage of mothers with less than 6 years of education was highest in Bangladesh (63.3%) and lowest in South Africa (2.1%). Nepal had the highest percentage of mothers with fewer than three living children (87.7%), whereas Tanzania had the lowest (27.8%). Access to improved drinking water sources was nearly universal except in Tanzania (42.6%), and improved latrine access was highest in South Africa (97.9%) and lowest in Tanzania (9.1%). Monthly incomes above $150 were most common in Brazil (97.6%) and nonexistent in Tanzania (0%). The WAMI score was highest in Brazil and South Africa (0.8) and lowest in Tanzania (0.2), whereas the EED score was highest in Peru (5.8) and lowest in Tanzania (4.1).

**Table 1:** General characteristics of the study subjects

Characteristics, *n* (%)	Bangladesh	Brazil	India	Nepal	Peru	South Africa	Tanzania
Male sex	108 (51.4)	89 (53.9)	105 (46.3)	122 (53.7)	105 (54.1)	120 (50.6)	105 (50.2)
Days of exclusive breastfeeding*	143.2 ± 42.7	93.7 ± 57.8	105.4 ± 42.9	92.5 ± 54.5	89.5 ± 61.3	38.6 ± 26.3	62.2 ± 35
Length-for-age Z score (first follow-up)*	−1.0 ± 1.1	−0.3 ± 1.3	−1.2 ± 1.3	−0.7 ± 1.1	−1.2 ± 1.0	−0.9 ± 1.1	−1.0 ± 1.1
Weight-for-age Z score (first follow-up)*	−1.1 ± 1.0	0.4 ± 1.0	−1.4 ± 1.2	−0.7 ± 1.1	−0.6 ± 1.1	−0.3 ± 1.0	−0.3 ± 1.1
Weight-for-length Z score (first follow-up)*	−0.5 ± 1.1	0.9 ± 1.3	−0.8 ± 1.2	−0.2 ± 1.3	0.4 ± 1.2	0.6 ± 1.3	0.9 ± 1.1
Length-for-age Z score (24 months follow-up)*	−2.0 ± 0.9	0 ± 1.1	−1.9 ± 1.0	−1.3 ± 0.9	−1.9 ± 0.9	−1.7 ± 1.1	−2.7 ± 1.0
Weight-for-age Z score (24 months follow-up)*	−1.6 ± 1.0	0.4 ± 1.2	−1.7 ± 0.9	−0.9 ± 0.9	−0.8 ± 0.9	−0.5 ± 1.0	−1.3 ± 1.0
Weight-for-length Z score (24 months follow-up)*	−0.8 ± 0.9	0.5 ± 1.4	−0.9 ± 0.9	−0.3 ± 0.9	0.3 ± 0.9	0.5 ± 1.0	0.1 ± 1.0
Maternal age (years)*	25.0 ± 5.0	25.4 ± 5.6	23.9 ± 4.2	26.6 ± 3.7	24.8 ± 6.3	27 ± 7.2	29.1 ± 6.5
Maternal height (cm)*	149.0 ± 5.0	155.1 ± 6.7	151.1 ± 5.2	149.7 ± 5.3	150.2 ± 5.5	158.7 ± 6.6	155.9 ± 5.9
Maternal educational level <6 years	133 (63.3)	22 (13.3)	80 (35.2)	59 (26)	44 (22.7)	5 (2.1)	75 (35.9)
Mother has less than 3 living children	160 (76.2)	113 (68.5)	157 (69.8)	199 (87.7)	111 (57.2)	141 (59.5)	58 (27.8)
Improved drinking water source	210 (100)	165 (100)	227 (100)	227 (100)	184 (94.9)	196 (82.7)	89 (42.6)
Improved latrine	210 (100)	165 (100)	121 (53.3)	227 (100)	66 (34)	232 (97.9)	19 (9.1)
Monthly income >$150	69 (32.9)	161 (97.6)	19 (8.4)	106 (46.7)	58 (29.9)	179 (75.5)	0 (0)
WAMI score*	0.6 ± 0.1	0.8 ± 0.1	0.5 ± 0.2	0.7 ± 0.1	0.5 ± 0.1	0.8 ± 0.1	0.2 ± 0.1
EED score*	5.2 ± 2.0	5.1 ± 2.3	4.8 ± 2.1	4.7 ± 2.0	5.8 ± 2.2	5.4 ± 2.3	4.1 ± 2.6

EED = environmental enteric dysfunction; WAMI = water/sanitation, assets, maternal education, and income.

* Mean ± SD.

**Figure 3.**
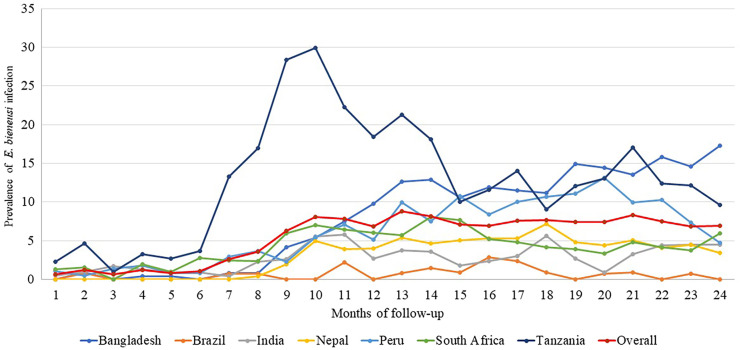
Site-specific prevalence of *Enterocytozoon bieneusi* infection by time.

Figure 3 presents the prevalence of *E. bieneusi* infection in children under 2 years in various geographical sites across different months. The overall prevalence ranges from 0.81% in month 1 to 17.84% in month 24, with the highest prevalence seen in months 9, 10, and 11. The highest prevalence was observed in Tanzania (with a maximum of 30.17% in month 10), followed by Bangladesh and Peru. The lowest prevalence was seen in Brazil and India.

#### Strength of association.

Table 2 presents the impact of *E. bieneusi* infection on three measures of growth in children: LAZ, WAZ, and WLZ. The data are presented for two groups: group A, which comprises children that had no *E. bieneusi* infection at any point during the study, and group B, which comprises children who became infected with *E. bieneusi* during the 24 months of follow-up. At baseline (first-month measurement), there were no significant differences in LAZ, WAZ, or WLZ between the two groups. However, at the end point (last-month measurement), significant differences were observed in all three measures. For LAZ, the infected group (group B) had a significantly lower score than the noninfected group (group A) (adjusted D-in-D = −0.53, 95% CI: −0.67 to −0.38; *P* <0.001), indicating that *E. bieneusi* infection was associated with reduced linear growth in children. For WAZ, the infected group (group B) also had a significantly lower score than the noninfected group (group A) (adjusted D-in-D = −0.38, 95% CI: −0.52 to −0.23; *P* <0.001), indicating that *E. bieneusi* infection was associated with reduced weight gain in children. For WLZ, the infected group (group B) had a significantly lower score than the noninfected group (group A) (adjusted D-in-D = −0.22, 95% CI: −0.38 to −0.06; *P* = 0.009), indicating that *E. bieneusi* infection was associated with reduced weight gain relative to linear growth in children. The study used a mixed-effect linear model to adjust for potential covariates such as copathogens, EED score, breastfeeding, maternal height and age, child’s sex, and WAMI index. The results suggest that *E. bieneusi* infection has a negative impact on child growth, particularly linear growth and weight gain.

**Table 2:** Growth and nutritional scores by infections with Enterocytozoon bieneusi*

Indicators	Baseline: First-Month Measurement			Endpoint: Last-Month Measurement			Adjusted D-in-D (95% CI)	*P*-Value (D-in-D)
	Group A	Group B	Adjusted Mean Difference (B − A) (95% CI)	Group A	Group B	Adjusted Mean Difference (B − A) (95% CI)		
LAZ	−0.82 ± 1.20	−1.00 ± 1.13	−0.01 (−0.14 to 0.12)	−1.35 ± 1.24	−2.06 ± 1.05	−0.13 (−0.24 to −0.03)	−0.53 (−0.67 to −0.38)	<0.001
WAZ	−0.58 ± 1.21	−0.66 ± 1.16	0.00 (−0.12 to 0.13)	−0.74 ± 1.25	−1.19 ± 1.04	−0.17 (−0.28 to −0.05)	−0.38 (−0.52 to −0.23)	<0.001
WLZ	0.08 ± 1.39	0.20 ± 1.37	0.02 (−0.12 to 0.17)	−0.07 ± 1.18	−0.16 ± 1.05	−0.13 (−0.24 to −0.01)	−0.22 (−0.38 to −0.06)	0.009

D-in-D = difference-in-difference; LAZ = length-for-age Z score; WAZ = weight-for-age Z score; WLZ = weight-for-length Z scores.

* Group A: At both baseline and endpoint, there was no infection with *E. bieneusi.* Group B: Noninfected at the first follow-up but became infected with *E. bieneusi* at least once during the 24 months of follow-up. The adjusted mean and adjusted D-in-D were estimated using a mixed-effect linear model at the site level after adjustment for the first principal component of copathogen, environmental enteric dysfunction score, number of children, days of exclusive breastfeeding, maternal height and age, child’s sex, and water/sanitation, assets, maternal education, and income (WAMI) score; the outcome variables were LAZ, WAZ, and WLZ. *Aeromonas*, *Bacteroides fragilis*, *Campylobacter jejuni/coli*, *Clostridium difficile*, Enteroaggregative *Escherichia coli*, Typical Enteropathogenic *Escherichia coli*, Shiga Toxin-producing *Escherichia coli*, Enterotoxigenic *Escherichia coli*, *Blastocystis hominis*, *Cryptosporidium*, *Giardia*, adenovirus 40/41, astrovirus, rotavirus, sapovirus, and norovirus were used as copathogens.

## DISCUSSION

For children enrolled in the MAL-ED birth cohort study, the current study assessed the prevalence of *E. bieneusi* infection and its association with various forms of childhood malnutrition. The prevalence of *E. bieneusi* infection showed significant variation among the different study sites (Figure 3). This study revealed that the overall prevalence of *E. bieneusi* infection displays an upward trend, suggesting a general increase in prevalence of infection over time in all the listed countries. Considering the prevalence of *E. bieneusi* infection across study sites, Tanzania has the most noticeable peak, suggesting a significant increase in infection during the middle of the period, notably from the 10th to the 15th month of follow-up, followed by Bangladesh and Peru. The lowest prevalence was credited to Brazil, followed by India and then Nepal. A definite rise in the prevalence of *E. bieneusi* infection was observed from the 9th month to the 24th month of follow-up, indicating that less hygienic weaning foods expose children to an increased risk of infection.^36^

This study used a mixed-effect linear model to enhance robustness by adjusting for country sites and to account for potential covariates, including copathogens, EED score, breastfeeding, maternal height and age, child’s sex, and the WAMI index. This allowed for a more comprehensive analysis of the relationship between *E. bieneusi* infection and child growth, while controlling for potential variables.^37^ The findings presented in this paper suggest that *E. bieneusi* infection is associated with reduced growth in children, particularly in terms of linear growth and weight gain.^32^ The results showed that there were no significant differences in LAZ, WAZ, or WLZ scores between group A and group B (the children in this group were infected by *E. bieneusi* over the period) at baseline. However, at the endpoint, significant differences were observed in all three measures, indicating the negative impact of *E. bieneusi* infection on child growth.^28^

The data showed a defined association between *E. bieneusi* and growth, but the causal role of *E. bieneusi* might not be conclusively established. A previous study showed that conditions like poor hygiene and sanitation affected the host predisposed to *E. bieneusi* infections.^10^ Additionally, results from the statistical association suggested that it is plausible that malnutrition could also have been a predisposing factor for *E. bieneusi* infection. Previous studies have also reported a link between parasitic infections, including *E. bieneusi* infections, and malnutrition in children.^32^ In low- and middle-income countries, parasitic infections are commonly found among children and can lead to chronic inflammation and decreased nutrient absorption, contributing to malnutrition.^7^ The findings of this study were in line with previous research and suggest that *E. bieneusi* infection might play a significant role in childhood malnutrition. However, the mechanisms through which *E. bieneusi* infection contributes to malnutrition are not fully understood and require further investigation. Additionally, the relationship between *E. bieneusi* infection and malnutrition is complex and may be influenced by other factors such as access to health services and adequate nutrition, as well as social and economic factors.^2^^–^^5^ Interventions to prevent and treat *E. bieneusi* infection, as well as to address other underlying factors, will be important in reducing the burden of childhood malnutrition.

Based on the results of this study, it is recommended that efforts be made to prevent and treat *E. bieneusi* infection to reduce the burden of childhood malnutrition. This may include interventions to improve hygiene and sanitation practices, as well as access to adequate nutrition and health services. It is also important to address other underlying factors that may contribute to childhood malnutrition, such as maternal nutrition and education, access to mass media, and involvement in income-generating activities.^4^^,^^38^^,^^39^ Further research is necessary to fully understand the mechanisms by which *E. bieneusi* infection is associated with malnourishment and inform effective prevention and treatment strategies. Moreover, the current recommendations for treating microsporidian infections are quite scant, with only two classes of drugs available: benzimidazoles and terpenes. Therefore, it is advisable to use these treatment options judiciously. Additionally, it is recommended that a multifaceted approach be taken, with consideration of the complex relationship between *E. bieneusi* infection, malnutrition, and other factors. This will require collaboration between public health, health services, and other relevant stakeholders.

The study used a birth cohort design, which followed individuals from birth over a long period of time, allowing for the examination of the development of outcomes over time. To increase the reliability of the results, standardized measures and protocols were used to assess *E. bieneusi* infection and children’s LAZ, WAZ, and WLZ. The study controlled for potential confounding factors, further improving the validity of the findings. This thorough approach allowed for a more accurate understanding of the relationship between *E. bieneusi* infection and childhood malnutrition. The study, which investigated the relationship between *E. bieneusi* infection and children’s LAZ, WAZ, and WLZ, had several limitations. First, the study population was restricted to a specific subgroup of newborns and their families because of the inclusion criteria and may not represent the general population. Second, although the study was conducted in eight different geographic locations, the results might not be generalizable to other populations or locations that might be reflected in the data, as the prevalence of *E. bieneusi* infection varied significantly within the cohort across different study sites. Finally, as an observational cohort study, establishing a causal association between growth and *E. bieneusi* infection was not feasible, given the ambiguity surrounding whether the infection preceded the outcomes, despite the apparent association observed.

## CONCLUSION

This study provides evidence that *E. bieneusi* infection is associated with reduced growth in children, particularly in terms of linear growth and weight gain. The findings underscore the importance of preventing and treating *E. bieneusi* infection in children, particularly in low- and middle-income countries where the infection is prevalent and children are at risk for poor growth outcomes. The study also highlights the need for public health interventions that address the broader determinants of child growth, such as malnutrition and poor sanitation. Further research is needed to elucidate rapid detection of parasites like *E. bieneusi* and the mechanisms underlying the negative impact of *E. bieneusi* infection on child growth and to develop effective interventions to mitigate this impact.
